# Uncommon presentation of morphea related to interferon beta in a patient with concomitant multiple sclerosis and chronic hepatitis C: A case report

**DOI:** 10.1002/ccr3.2971

**Published:** 2020-05-26

**Authors:** Mrinal Gupta, Paul S. Yamauchi, Martine Bagot, Jacek Szepietowski, Shibani Bhatia, Torello Lotti, Mohamad Goldust

**Affiliations:** ^1^ Treatwell Skin Centre Jammu India; ^2^ Dermatology Institute and Skin Care Center Santa Monica California USA; ^3^ Division of Dermatology David Geffen School of Medicine at University of California Los Angeles California USA; ^4^ AP‐HP Dermatology Department Saint‐Louis Hospital INSERM U976 Université Paris Diderot‐Paris VII Sorbonne Paris Cité Paris France; ^5^ Department of Dermatology, Venereology and Allergology Wroclaw Medical University Wroclaw Poland; ^6^ Department of Dermatology, Venereology and Leprosy Kasturba medical college Manipal Manipal Academy of Higher Education Manipal India; ^7^ University of Studies Guglielmo Marconi Rome Italy; ^8^ Mazandaran University of Medical Sciences Sari Iran; ^9^ University of Rome G. Marconi Rome Italy; ^10^ Department of Dermatology University Medical Center Mainz Mainz Germany; ^11^ Department of Dermatology University Hospital Basel Basel Switzerland

**Keywords:** interferon beta‐1b, morphea, multiple sclerosis

## Abstract

Recombinant interferon beta‐1b is one of the treatment options of multiple sclerosis (MS). Insertional biologics that are used in the treatment of MS may lead to skin adverse effects, for example, morphea.

## Graphical abstract

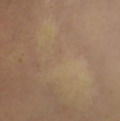

## 1 INTRODUCTION

IFNβ‐1b is a cytokine, which belongs to interferon group, and is used in management of multiple sclerosis. It is administered only as an injectable medication. Cutaneous adverse effects with IFNβ‐1b have a wide array of presentations and are commonly seen with subcutaneous route.^1^ They usually appear within one month of treatment irrespective of the frequency of administration and are usually seen within six months of initiation of treatment. Cutaneous reactions are more commonly seen in women. They include local site reactions like skin necrosis, lipoatrophy and systemic cutaneous disorders like psoriasis, systemic lupus erythematosus, and dermatomyositis which have been reported. Mild adverse effects usually do not require cessation of treatment, whereas necrosis is seen in around 5% of patients and may require discontinuation of the treatment.^1^, ^2^ Morphea is a type of localized scleroderma, which is three times more commonly seen in women than men.^3^, ^4^ Here we report a case with concomitant multiple sclerosis and chronic hepatitis C who presented with cutaneous woody indurated plaques which were histologically consistent with diagnosis of morphea at the sites of IFN‐b1a injections.

## 2 CASE REPORT

**Figure 1:**
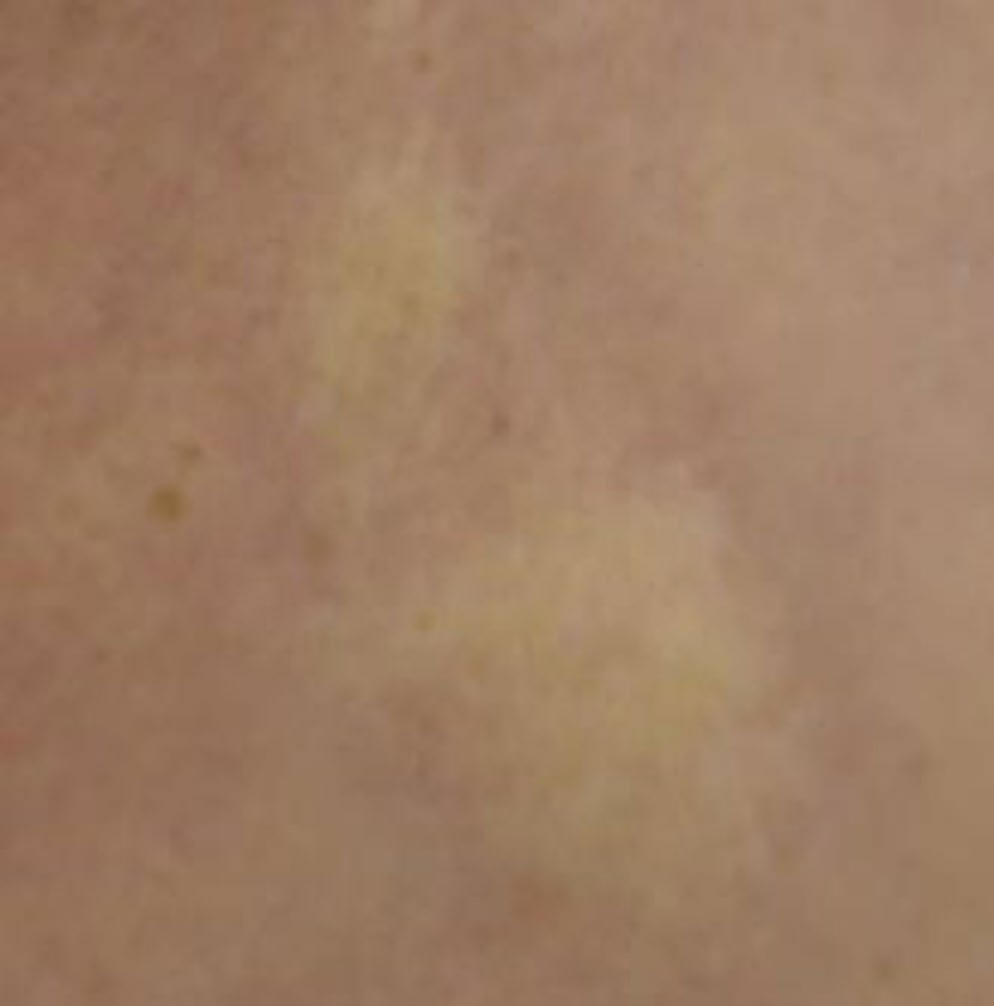
Indurated, depressed bound‐down plaques involving anterior thighs

**Figure 2:**
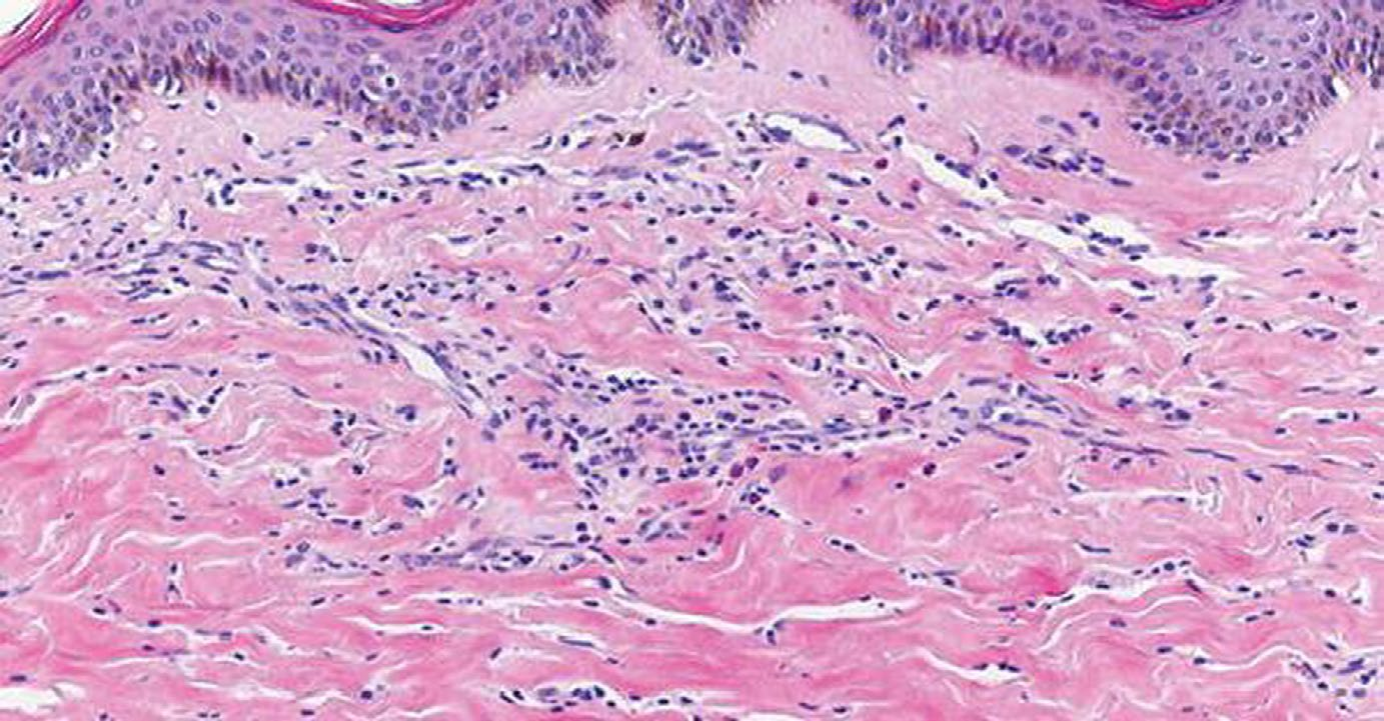
Thickening of collagen bundles and a lymphoplasmacytic infiltrate in the deep reticular dermis

A 64‐year‐old patient, a known case of multiple sclerosis, was on treatment with injection IFNβ‐1b (Avonex with dosage of 30 mcg IM qWk) for the same for 8 years. She came with complaints of tightness of skin with discoloration, which started two years after starting treatment with IFNβ‐1b. The lesions were exclusively present over the injection sites. The lesions gradually progressed to become more firm, depressed, and hyperpigmented (Figure 1). She was advised to discontinue the IFNβ‐1b therapy and took treatment with topical steroid and calcitriol for two years before presenting to us. The induration progressed despite discontinuation of interferon injections. The patient also had a history of chronic hepatitis of more than 30 years duration and was treated with ribavirin and sofosbuvir since the diagnosis. Since the lesions were progressing, we decided to perform a skin punch biopsy for histopathology from the right thigh. It revealed thickening and generalized homogenization of collagen bundles, which extended into the subcutaneous fatty tissue, near‐complete absence of adnexal structures, along with a lymphoplasmacytic infiltrate involving the deep reticular dermis (Figure 2). These microscopic findings were consistent with inflammatory stage of morphea, thus confirming the diagnosis of morphea at the sites of injection of interferon.

## 3 COMMENT

Morphea is characterized by excessive collagen deposition leading to thickness of the dermis, subcutaneous tissues, or both.^5^, ^6^ IFNβ‐1b acts by balancing the expression of both pro‐ and anti‐inflammatory mediators in the brain tissue, thereby reducing the number of inflammatory cells crossing the blood‐brain barrier. IFNβ‐1b therapy has been reported to reduce neuronal inflammation and also increase the nerve growth factor production and thereby increasing the survival of the neurons. Adverse reactions of interferon beta‐1b include flu‐like illness presenting with fever, headache, chills, tiredness, increased sweating, muscle pains, and malaise. Few cases of sclerosing skin disorders after treatment with tumor necrosis factor inhibitors and IFNβ‐1b have been reported. Most recently, IFN‐b1a has been reported to be a possible trigger for development of systemic sclerosis in patients of multiple sclerosis.^7^, ^8^ Our case had clinical and histological manifestations consistent with morphea which developed at the injection sites after beginning of IFN‐b1a treatment. The patient presented with localized scleroderma‐like changes which were localized only to the injection sites with no other clinical features indicating limited or diffuse systemic sclerosis. Bezalel et al described a patient of multiple sclerosis who presented with a woody hard induration which was clinically and histologically consistent with morphea which appeared at the sites of IFN‐β1a injections and stated that the increased use of biologics in the management of various conditions and the potential interaction between these treatment modalities and patients' inflammatory mediators may increase the risk of cutaneous side effects.^9^ Their patient had no comorbidity in contrary to our case that had chronic hepatitis C which was treated with ribavirin. Waki et al evaluated a case of localized scleroderma (LS) occurring after initiation of sofosbuvir and ribavirin in chronic hepatitis C and stated that the disease itself along with the drugs affected immune activation leading to the development of LS.^10^ Telakis & Nikolaou studied a case of morphea in a patient of chronic hepatitis C and postulated that a direct causal relationship between HCV infection and morphea cannot be suggested owing to the small number of cases reported in literature. Routine evaluation of HCV infection in patients of morphea might help in strengthening this correlation. The overall effect of antiviral drug therapy in HCV patients having morphea requires more study.^11^ Although disease severity and treatment of HCV may affect immune system and increase the probability of morphea development, our case demonstrated morphea lesion at the injection site which can be more likely attributed to IFNβ‐1b injections. As most of biological are self‐injecting therapies, it becomes difficult to assess if these cutaneous adverse effects are caused by vascular damage at injection sites or a treatment‐induced change in the cytokine profile of the patient. Hugle et al described and analyzed the clinical features of sclerosing skin diseases in cases with MS and demonstrated that sclerosing skin diseases can develop in the course of treatment of MS. It has been observed that sclerosing disorders appear earlier in patients with MS which suggests possible genetic predisposition or an IFN‐associated stimulus.^12^

## CONFLICT OF INTEREST

None declared.

## AUTHORS CONTRIBUTIONS

All authors have read and approved the manuscript, and ensure that this is the case. MG: involved in patient follow‐up and management and final approval of the version to be published. PSY: involved in patient management and writing article, drafted the work, and substantively revised it. MB: involved in patient follow up and management, drafted the work, and substantively revised it. JS: involved in patient management and drafting the manuscript. SB: involved in patient follow‐up and management, drafted the work, and substantively revised it. TL: involved in patient follow‐up and management, drafted the work, and substantively revised it. MG: involved in patient follow‐up and management, drafted the work, and substantively revised it.
